# Molecular subdivision of the marine diatom *Thalassiosira rotula* in relation to geographic distribution, genome size, and physiology

**DOI:** 10.1186/1471-2148-12-209

**Published:** 2012-10-26

**Authors:** Kerry A Whittaker, Dayna R Rignanese, Robert J Olson, Tatiana A Rynearson

**Affiliations:** 1 Graduate School of Oceanography, South Ferry Road, University of Rhode Island, Narragansett, RI 02882, USA; 2 Biology Department, Woods Hole Oceanographic Institution, Woods Hole, MA, 02543, USA

**Keywords:** Phytoplankton, Phylogeography, Dispersal, Physiology, Intraspecific diversity

## Abstract

**Background:**

Marine phytoplankton drift passively with currents, have high dispersal potentials and can be comprised of morphologically cryptic species. To examine molecular subdivision in the marine diatom *Thalassiosira rotula,* variations in rDNA sequence, genome size, and growth rate were examined among isolates collected from the Atlantic and Pacific Ocean basins. Analyses of rDNA included *T. gravida* because morphological studies have argued that *T. rotula* and *T. gravida* are conspecific.

**Results:**

Culture collection isolates of *T. gravida* and *T. rotula* diverged by 7.0 ± 0.3% at the ITS1 and by 0.8 ± 0.03% at the 28S. Within *T. rotula*, field and culture collection isolates were subdivided into three lineages that diverged by 0.6 ± 0.3% at the ITS1 and 0% at the 28S. The predicted ITS1 secondary structure revealed no compensatory base pair changes among lineages. Differences in genome size were observed among isolates, but were not correlated with ITS1 lineages. Maximum acclimated growth rates of isolates revealed genotype by environment effects, but these were also not correlated with ITS1 lineages. In contrast, intra-individual variation in the multi-copy ITS1 revealed no evidence of recombination amongst lineages, and molecular clock estimates indicated that lineages diverged 0.68 Mya. The three lineages exhibited different geographic distributions and, with one exception, each field sample was dominated by a single lineage.

**Conclusions:**

The degree of inter- and intra-specific divergence between *T. gravida* and *T. rotula* suggests they should continue to be treated as separate species. The phylogenetic distinction of the three closely-related *T. rotula* lineages was unclear. On the one hand, the lineages showed no physiological differences, no consistent genome size differences and no significant changes in the ITS1 secondary structure, suggesting there are no barriers to interbreeding among lineages. In contrast, analysis of intra-individual variation in the multicopy ITS1 as well as molecular clock estimates of divergence suggest these lineages have not interbred for significant periods of time. Given the current data, these lineages should be considered a single species. Furthermore, these *T. rotula* lineages may be ecologically relevant, given their differential abundance over large spatial scales.

## Background

Photosynthetic organisms in terrestrial and marine habitats contribute to global primary production in roughly equal proportions [1,2]. For most terrestrial photoautotrophs, species distributions occupy two spatial dimensions and vary over relatively long time scales [3-6]. In marine habitats, most primary producers are unicellular phytoplankton, smaller than 200μm, that drift with tides and currents. Marine phytoplankton differ from their terrestrial counterparts in that these tiny organisms have higher dispersal potentials, the ability to occupy three spatial dimensions, and species distributions that can vary over time scales of days to weeks [7-9].

Within the phytoplankton, diatoms are a particularly important class of algae. These commonly-occurring organisms generate over 20% of global primary production, and thus play a key role in driving global biogeochemical cycles [10]. They are found in almost all aquatic habitats, are comprised of an estimated 200,000 species [11] and yet only arose in the early Mesozoic (~ 250 mya) [12]. Diatom species have long been identified based on their ornate siliceous frustule, or cell covering, and recently, based on DNA sequence variation. Morphological differences in the frustule are often subtle and with the advent of scanning electron microscopy, additional morphological features were visible and many new species were described [13]. More recently, ribosomal DNA sequence variation has been used to identify morphologically identical, or cryptic, species [14-20]. Thus far, no clear barcoding gene, such as Cox1, has been identified for classifying diatom species [21,22].

Testing the biological species concept using diatoms poses a significant challenge because it has proven difficult to consistently control sexual reproduction in the laboratory. For those species where sexual reproduction can be controlled, there appears to be a relationship between reproductive incompatibility and compensating base changes in the stem regions of ITS2 secondary structures [15,23-25]. Although this same relationship has not been explicitly observed for the ITS1, it has been argued that the two genes do not evolve independently of one another, and thus their secondary structures are similarly conserved [26]. In laboratory experiments with the diatom genus *Sellaphora*, interbreeding was successful between individuals with 7.3% divergence at the ITS1 and ITS2, but not between those with 10% divergence [25]. Similarly, reproductive isolation has been observed between species of the genus *Pseudo-nitzschia* that diverged by 2.4% at the ITS1 and ITS2. Morphologically cryptic diatom species have also been identified by 0.5% sequence divergence at the 28S rDNA [18-20,27]. Furthermore, diatom lineages have been shown to exhibit differences in genome size [28,29], suggesting that polyplidization may play a role in driving cryptic diatom speciation, as is commonly observed in plants [30].

The identification of morphologically cryptic species has led to the question of whether cosmopolitan species are truly globally distributed or whether these morphospecies are instead divided into multiple species with distinct biogeographic ranges. For example, the diatom *Skeletonema costatum* was once thought to be a “super” species based on its ability to thrive and even dominate phytoplankton communities in an exceptionally broad range of environments [31]; it was recently shown to consist of several different species [16,18-20,32] that may each have unique geographic distributions [32]. Similarly, geographic differentiation has been shown for the harmful algal bloom-forming genus *Pseudo-nitzschia*[33,34]. Aside from these few examples, it is unknown whether most ecologically important diatoms with cosmopolitan distributions are true species or whether they are comprised of multiple cryptic species. Furthermore, understanding the genetic relationships within ecologically important species or species complexes from geographically disparate regions has become an important issue in light of climate change and the novel selection pressures that may result [35-37].

It has been challenging to describe general patterns of species division in diatoms because previous studies used different methods, focused on different species, and often sampled few isolates from a restricted spatial scale. We focused on identifying genetic subdivision in the diatom morphospecies *Thalassiosira rotula* by simultaneously examining variation in rDNA sequences, physiology, and genome size from isolates collected from around the globe. *Thalassiosira rotula* is a commonly-occurring diatom that can dominate phytoplankton assemblages across diverse marine habitats and hydrographic environments (eg. [31,38-46]). Here, cells were collected along a transect in the Eastern North Pacific and their rDNA (18S, ITS1, 28S) compared with isolates collected from the Pacific, the Atlantic, and Mediterranean Sea to determine the geographic distribution of rDNA sequence variants. Growth rates among isolates were used to determine the relationship between molecular and physiological diversity. Variation in genome size among isolates was measured as recent work indicated that differences in DNA content may identify cryptic species [28,29]. Morphological studies suggested that *T. rotula*[47] and *T. gravida*[48] are likely a single species [49,50]. To test this hypothesis, we determined rDNA sequence variation among culture collection isolates of *T. rotula* and *T. gravida*. By examining isolates collected from around the world, we were able to identify sufficient genetic differences between *T. rotula* and *T. gravida* to warrant their continued description as distinct species and to identify genetic subdivision within *T. rotula* and its correspondence to geographic location, physiological variation and differences in genome size.

## Methods

### Isolates

Cells of the diatom morphospecies *Thalassiosira rotula/ gravida* were collected from 8 locations in the Eastern and Western Pacific and Western Atlantic between 2007 and 2009 (Table 1). Culture collection isolates of *T. rotula* and *T gravida* from an additional 7 locations were also obtained (Table 1). For all field samples (sites 1–4, 7, 8, and 13), surface water was passed through a 20μm mesh net. Single cells or short chains were isolated from the >20μm size fraction using a stereomicroscope (Olympus SZ61) washed in sterile seawater three times, and transferred to 1 mL sterile Sargasso seawater amended with f/20 nutrients [51]. Cells were incubated at 8°C (site 13) or 14°C (all other sites) and on a 12:12-h light:dark cycle at 90 μmol photons m^-2^ s^-1^ for approximately two weeks. Live cells were then examined using microscopy to record growth and detect contamination. When cell density reached ~1000 cells/ml, 20μl of each isolate were transferred to fresh f/20 media to maintain growth. Remaining cells were harvested onto a 1.2 μm filter and frozen at −80°C until further analysis. Culture collection isolates from NCMA (National Center for Marine Algae and Microbiota, formerly CCMP), CCAP (Culture Collection of Algae and Protozoa), and Japan were grown at 4°C (sites 14, 15) or 14°C (all others) and maintained in exponential growth; cells were filtered and frozen as described above. Genomic DNA of both field and culture collection isolates was extracted using the DNeasy Plant Mini Kit or the DNeasy 96 Plant Kit (Qiagen, Inc.).

**Table 1:** Description of site and isolates collected, including isolation success and genes sequenced from each site

	**Site**	**Region**	**Origin**	**Date Collected**	**No. cells isolated (survived)**	**ITS1 sequences (#)**	**18S and 28S Sequences (#)**	**Lineage 1**	**Lineage 2**	**Lineage 3**	**Physiology**
											
**a.)**	1	North Pacific	Queen Charlotte Islands, Canada 51.75°N, 131°W	5/17/2007	21 (18)	10		3	6	1	
	2	North Pacific	Vancouver Island (A), Canada 49.65°N, 127.44°W	5/13/2007	48 (41)	10	1	10			1
	3	North Pacific	Vancouver Island (B). Canada 48.87°N, 125.89°W	5/13/2007	32 (30)	10	1	10			1
	4	North Pacific	Puget Sound, USA 47.74°N, 122.42°W	5/12/2007	56 (56)	10	1		10		
	5	North Pacific	La Jolla, USA 32.85°N, 117.25°W (CCMP1018)	1968	NA	1	1	1			1
	6	North Pacific	Seto Inland Sea, Japan 34.16°N, 133.33°E	2/2007	NA	5	1			5	1
	7	North Atlantic	Martha’s Vineyard Coastal Observatory, USA 41.45°N, 70.56°W	11/3/2008	17 (15)	10				10	
	8	North Atlantic	Narragansett Bay, URI 41.53°N, 71.38°W	10/10/2008	24 (24)	10				10	
					1/8/2009	24 (21)	10	1			10	
				2/9/2009	48 (45)	10				10		
				6/26/2009	4 (2)	2						
	9	North Atlantic	Oban, Scotland 56.57°N, 5.43°W (CCAP1085_20)	2008	NA	1	1			1		
	10	Mediterranean Sea	Gulf of Naples, Italy 40.95°N, 14.25°E (CCAP1085_21)	2008	NA	1	1			1	1	
	11	Mediterranean Sea	Gulf of Naples, Italy 40.49°N, 14.15°E (CCMP1647)	11/12/2008	NA	1	1			1		
	12	Mediterranean Sea	Gulf of Naples, Italy 40.75°N, 14.33°E (CCMP3264)	1993	NA	1	1			1	1	
**b.)**	13	North Atlantic	Iceland 60.92472°N, 27.005833°	5/22/2008	48 (29)	10	3					
	14	North Atlantic	Tromso, Norway 69.66°N, 18.96°E (CCMP986, 987)	8/1/1978	NA	2	1					
	15	Southern Ocean	McMurdo Sound77.83°S 163.00°E, Antarctica (CCMP1463, CCMP1462)	2/1/1991	NA	2	2					

**Figure 1:**
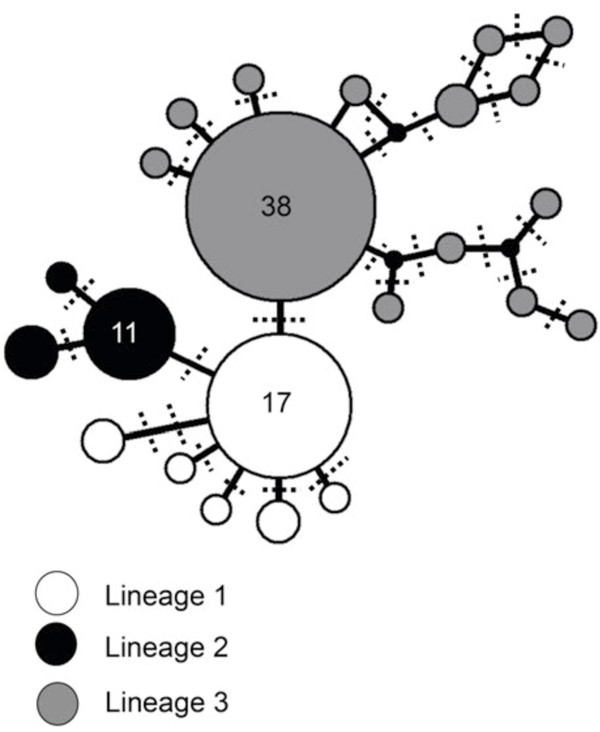
**Network Analysis.** Network analysis representing the most parsimonious relationship between sequence variants, separated by single base pair mutations (dotted lines). From 92 isolates, three lineages were defined (those with more than 10 isolates/sequence type, plus their most closely associated sequence types). Each color represents a lineage, each circle represents a sequence variant, and its size indicates the number of isolates comprising each sequence variant. Numbers inside each node indicate the number of isolates comprising that sequence type. All other nodes represent 1, 2, or 4 isolates, scaled to size.

Site numbers refer to map featured in Figure 1. Isolation number or isolation success number was not available for culture collection strains, nor for those isolated in Japan. Culture collection accession numbers are indicated in the “Origin" column. a) All *T. rotula* isolates, including number classified as 1, 2, or 3, number of isolates included in physiological experiments. b) Description of *T. gravida* isolates.

### Ribosomal DNA sequencing and analysis

To quantify genetic variation among isolates, three regions of the ribosomal DNA (rDNA) were sequenced: the small subunit (18S), the D1 hypervariable region of the large subunit (28S), and the internal transcribed spacer region I (ITS1). To amplify each rDNA region, a reaction mixture containing ~5ng DNA, 0.1 mmol L^-1^ dNTPs (Bioline), 0.05 U μL^-1^ Accuzyme DNA polymerase (Bioline), 1X buffer (Bioline), and 0.5 μmol L^-1^ each of forward and reverse primers was used. Using polymerase chain reaction (PCR), the ITS1 was amplified using a newly-designed primer specific to *T. rotula* and *T. gravida*: ROTIIR GTCACAGTCCAGCTCGCCACCAG and primer 1645F [52]. The PCR consisted of a 2 min denaturation step at 95°C, 36 cycles of 94°C for 30 s, 62°C for 30 s, and 72°C for 1 min followed by a 10 min extension at 72°C. The ITS1 was amplified from 106 isolates, including 10 from each field sample. The 18S was amplified from 16 of those isolates using universal 18SA and 18SB primers [53]. The PCR consisted of a 2 min denaturation step at 95°C, 33 cycles of 94°C for 20 s, 55°C for 60 s, and 72°C for 2 min and 10 min at 72°C. The D1 region of the 28S was also amplified from 16 isolates using the forward primer, 28SF: ACCCGCTGAATTTAAGCATA, and reverse primer, 28SR: ACGAACGATTTGCACGTCAG [54], at the same thermocycling conditions as the 18S PCR. All sequencing was performed on an ABI 3130xl (Applied Biosystems). Both strands of the ITS1 were sequenced to completion using primers 1645F and RotIIR. For 18S rDNA genes, both strands were sequenced to completion using primers 18SA and 18SB [53], 18SC2, 18SE2, and 18SF3 [55], and 18SD [56]. 28S was sequenced using 28SR and 28SF, respectively. All sequences are available in Genbank (accession numbers JX069320-JX069349 and JX074825-074930).

Sequences were assembled using SeqMan II 3.61 (DNASTAR, Inc.), and aligned using Clustal W [57] in Mega4 [58]; boundaries of the ITS1 were determined through alignment with Genbank accession EF208798. For ITS1, 18S, and 28S, sequence divergence was calculated using a two-parameter model of nucleotide substitution using Mega4 [58,59]. Significant differences in rDNA sequence variation between *T. rotula* and *T. gravida* were determined using analysis of molecular variance (AMOVA) with 1000 permutations. To examine relationships among *T. rotula* ITS1 variants*,* a network was generated using the median joining algorithm in Network 4.5.1.6 (Fluxus Technology Ltd.) and tested for statistical significance using AMOVA with 1000 permutations. Significant differences in within-lineage diversity were determined using the population differentiation algorithm [60] run with 1000 steps of the Markov Chain, 1000 dememorization steps, and an alpha of 0.05. All AMOVA and population differentiation analyses were conducted using Arlequin v. 3.11 [61]. The RNAalifold webserver ( http://rna.tbi.univie.ac.at/cgi-bin/RNAalifold.cgi) was used to construct the predicted secondary structure of the consensus ITS1 sequence from the Clustal W alignment of all isolates, using the minimum free energy and partition function, avoiding isolated base pairs [62].

A relaxed molecular clock with an uncorrelated lognormal model was used to generate divergence times of *Thalassiosira* species (Table 2) and *T. rotula* lineages in BEAST v1.4.6. The most frequently occurring sequence type in each *T. rotula* lineage was chosen for analysis. Trees were generated using the last 5,000 generations of a Markov chain-Monte Carlo analysis run for 1 x 10^6^ generations. To obtain molecular clock estimates, we used a fossil-calibrated, 18S divergence time of 11Mya for *T. aestevalis* and *T. anguste-lineata*[12]*.* This divergence time was forced on the *T. aestevalis* and *T. anguste-lineata* ITS1 divergence to obtain an estimated ITS1 mutation rate for the remaining *Thalassiosirids* compared here.

**Table 2:** ***Thalassiosira *spp. and Genbank accession numbers used in phylogenetic analysis**

**Species**	**Accession no.**
*T. pseudonana*	EF208793
*T. guillardii*	EF208788
*T. weissflogii*	FJ432753
*T. anguste-lineata*	EF208800
*T. aestivalis*	EF208797
*T. oceanica*	EF208795
*T. punctigera*	EF208796

To examine intra-individual variation in the multi copy ITS1, PCR amplicons from three individuals representing the three most dominant sequence types were ligated into the pCR2.1 vector, and chemically transformed into TOP10 one-shot competent *Escherichia coli* cells using the TOPO TA Cloning Kit (Invitrogen, Inc.). ITS1 inserts were amplified from cloned *E. coli* using the Illustra Templiphi Kit (GE) and 20 inserts from each isolate were sequenced as described above. Intra-individual ITS1 variants were added to the network analysis generated by Network 4.5.1.6 (Fluxus Technology Ltd.). Difference of sums of squares and probabilistic divergence measures in Topali v.2 were used to measure putative recombination break points among intra-individual ITS1 sequence types using a step size of 5, window size of 100, and 500 threshold runs. Additional tests for recombination were conducted in RDP3, using a UPGMA-based pairwise scanning approach [63].

### Physiological variation

Isolates of *T. rotula* collected from the Mediterranean Sea (CCMP1647, site 11), from the coast of Vancouver Island (VIA, site 2), and from the Seto Inland Sea (SIS, site 6) were made axenic to remove contaminating bacteria. Sterile glass tubes were prepared with 4.5 mL sterile f/2, 10 μL sterile bacterial test medium (Bacto TM) (5 g L^-1^ Bacto-peptone and 5 g/L malt extract), ~ 1000 cells of each isolate and different volumes of sterile antibiotic mix (50–400 μL) containing 0.1 g L^-1^ Penicillin G (Potassium salt), 0.0025 g L^-1^ dihydrostreptomycain sulfate, and 0.005 g L^-1^ gentamyacin. At 72, 96 and 120 hours, 5–50 μL of culture were subcultured into 4 mL of sterile f/2 media at 14°C with a 12:12 light:dark cycle and routinely tested for bacterial contamination using Bacto TM.

The resulting three axenic isolates (CCMP1647, VIA, and SIS) as well as three additional xenic isolates (CCMP3264, VIB, and CCAP1085_21, sites 12, 3, and 10 respectively) were incubated at three temperatures (4, 10, and 17.5°C) on a 12:12 h light:dark cycle at two light conditions (50 μmol and 112 μmol photons m^-2^ s^-1^). Cultures were allowed to acclimate until no differences in maximum growth rate were observed between transfers. Maximum acclimated growth rates were determined following Rynearson and Armbrust [64] and Brand [65]. Briefly, the in vivo fluorescence of semi-continuous batch cultures was measured daily using a 10AU Field Fluorometer equipped with the in vivo chlorophyll optical kit (Turner). The maximum acclimated growth rate for each isolate was determined by regressing the change in the log of fluorescence over time and testing the equality of slopes from at least three serial cultures (α = 0.05). If slopes of serial growth curves were homogenous, the average regression coefficient was used to estimate the common slope, which represented the average acclimated growth rate. To test for differences in growth rate between axenic and xenic strains, triplicate cultures of both axenic and xenic VIA were grown at 10°C, 100 μmol photons m^-2^ s^-1^. Analysis of variance (ANOVA), nested ANOVA, Sheffe’s Test [66], and the Tukey multiple comparison test [67] were used to determine the significance of differences among isolates at different temperatures and light levels, and between axenic and xenic growth rates. For each isolate, the diameter of 30 cells was measured using an E800 microscope at 20X (Nikon). One-way ANOVA was used to determine the significance of differences between cell size across all temperature and light conditions. Alpha was set to 0.05 for all statistical tests.

### Relative genome size

Five isolates were chosen to examine relative differences in genome size using flow cytometry: VIA (site 2), VIB (site 3), SIS (site 6), CCAP1085_21 (site 10), and CCMP3264 (site 12). To ensure that cells in both G1 and G2 phases were present in each sample, cells were grown in continuous light (20°C), 60 μmol photons m^-2^ s^-1^. For each isolate, triplicate cultures were harvested at mid-exponential phase by centrifugation (15 min at 112 rcf). Cells were injected into 10 ml of 100% methanol at 0°C using a 1 in, 22 gauge syringe needle, incubated at 4°C for ≥ 1 h to extract chlorophyll *a*, centrifuged three times for 15 min at 112 rcf, washed each time with 5 ml PBS (137 mM NaCl, 2.7 mM KCl, 10.4 mM Na2HPO4·H2O, 1.8 mM KH2PO4, pH = 7.4) and resuspended in 3 ml PBS at >1000 cells ml^-1^. Cellular DNA was stained with DAPI for ≥ 20 min. A 50 μL suspension of fluorescent latex beads (UV-excited, 6 μm diameter; Invitrogen) were added to each sample as an internal standard.

Flow cytometry was conducted using a benchtop version of the Imaging FlowCytobot (IFCB) [68] with a 375nm, 16 mW UV laser (CUBE™375-16C, Coherent, Inc.) and a beam spot approximately 10 μm high. Dichroic and bandpass filters directed blue light (DAPI fluorescence, 425–475 nm) to the fluorescence PMT (HC 120-05M, 10 MHz preamp, Hamamatsu used for triggering) and UV light (<400 nm) to the light scattering PMT. Sheath flow (0.2 μm filtered distilled water) was gravity-driven through the laser beam at a velocity of ~ 2 ml min^-1^. A digitizer (model AD2100-14, Chase Scientific), operating at 6 samples per μsec, recorded complete traces of the PMT response for each cell. Cell debris and clumps were removed from the dataset using images captured with each event and custom Matlab scripts, leaving only beads or well-defined *T. rotula* cells. Next, distributions of integrated fluorescence of triplicate *T. rotula* cultures for each isolate were combined and the cell data normalized to internal standard bead fluorescence. Mode values for the G1 peaks were determined from the bead-normalized distributions after smoothing the data (fastsmooth.m, width 10; T. C. O'Haver, pers. comm).

To investigate relationships between cell size and genome content, cell volume of each isolate analyzed using flow cytometry was determined by measuring cell diameter and length for 30 cells isolate^-1^ using an E800 microscope at 20X (Nikon). The significance of differences in average cell volume among strains was determined using ANOVA.

## Results

### rDNA variation

Culture collection isolates of *T. gravida* and *T. rotula* were not significantly different (p>0.05) at the 18SX, but diverged significantly (p<0.05) from each other at the ITS1 (7 ± 0.3%) and 28S (0.8 ± 0.03%). Of the 97 field isolates analyzed, 10 had 28S and ITS1 sequences that were identical (100%) to *T. gravida* culture collection isolates identified by taxonomists (Table 1b) and thus were designated as *T. gravida*. rDNA sequences of the remaining field isolates were identical (100%) at the 28S and 99-100% similar at the ITS1 to *T. rotula* culture collection isolates identified by taxonomists (Table 1a) and thus were designated as *T. rotula.*

**Figure 2:**
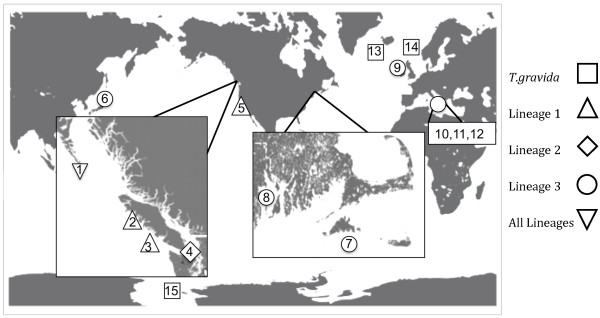
**Sample Map.** Map of global sample locations from where *T. rotula* isolates were collected. Numbers correspond to location and sample information given in Table 1.

Of 92 global *T. rotula* isolates, 23 unique ITS1 sequences were detected with an average sequence divergence of 0.6 ± 0.3%. Twenty sequences were relatively rare, identified in fewer than 4 isolates. Of these, sixteen sequences were identified just once, three sequences were identified twice, and one sequence was identified in four isolates. Three sequences (Table 3, sequences 1–3) were relatively abundant (identified in >10 isolates). The median joining network of all sequences was significant (AMOVA p<0.001), and was used to define three distinct lineages corresponding to the three most abundant sequences, and closely branching but rare sequences (Table 3, Figure 1). Lineage 1 was comprised of six sequences and was dominated by sequence 1, identified in 17 isolates (Table 3). All lineage 1 isolates originated from the coastal North Pacific (Figure 2, sites 1–3 and 5). Lineage 2 was comprised of three sequences and was dominated by sequence 2, identified in 11 isolates (Table 3). Lineage 2 isolates were sampled from Puget Sound (Figure 2, site 4) with the exception of six isolates sampled from coastal North Pacific waters (Figure 2, site 1). Lineage 3 was sampled from waters throughout the global ocean (Figure 2, sites 6–12) and consisted of 14 sequence types. Lineage 3 was dominated by sequence 3, identified in 38 isolates (Table 3).

**Table 3:** **ITS1 variation among 76 isolates of *Thalassiosira rotula *collected from around the globe**

**Lineage**	**Sequence ID**	**n**	**Origin**	**Site #**	**BP Position**								
					**41**	**44**	**50**	**82**	**95**	**108**	**149**	**200**	**201**
1	1	17	Vancouver Island (A and B)	2,3	A	G	C	C	C	A	-	G	G
1	4	2	Vancouver Island (A and B)	2,3	-	-	-	-	A/C	A/T	-	-	-
1	5	2	Queen Charlotte Islands, La Jolla, CA	1,5	-	-	-	-	-	-	C	-	-
2	2	11	Puget Sound, Queen Charlotte Islands	4,1	G/A	-	-	-	-	-	-	-	A
2	6	4	Puget Sound	4	G	-	-	-	-	-	-	-	G/A
3	3	38	Martha’s Vineyard, Narragansett Bay, Japan, Mediterranean, Scotland	6,7,8, 9,10,12	-	-	-	T	-	-	-	T	-
3	7	2	Mediterranean and Narragansett Bay	11,8	-	A	C/T	T	-	-	-	T	-

This table includes only those sequences represented by >1 isolate. No singleton sequences are displayed. Base pairs indicated with a slash (eg. A/C) indicate ambiguous signals in sequencing, and thus the possible presence of two alleles at that position.

**Figure 3:**
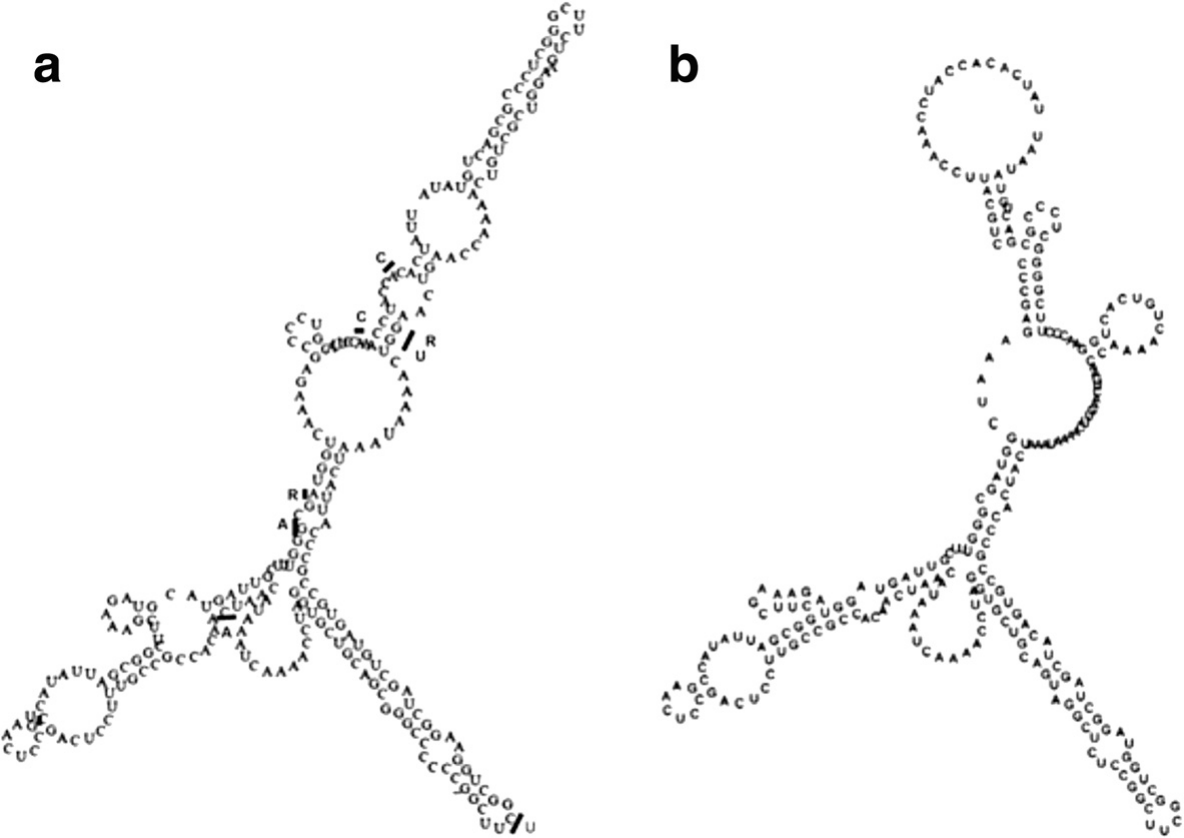
**ITS1 rDNA Folding Structures. a**. Predicted RNA folding structure of ITS1 in *T. rotula* based on consensus sequences of all global isolates. Bars and letters represent alternate base pairs seen in sequence variants. **b**. Predicted RNA folding structure of ITS1 in *T. gravida* based on consensus sequences of all isolates from Iceland and culture collections.

The predicted folding structure of the *T. rotula* ITS1 revealed no compensatory base changes among isolates either within or between lineages (Figure 3a). Most mutations occurred in loop regions. Only two mutations (G/A, G/U) occurred in adjacent positions along a stem region, but displayed no corresponding compensatory changes. *T. gravida* and *T. rotula* exhibited different predicted ITS1 folding structures (Figures 3a and 3b).

**Figure 4:**
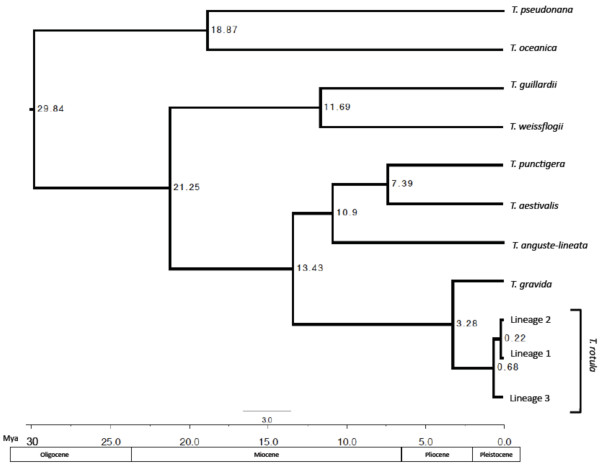
**Phylogenetic Analysis.** Bayesian analysis of divergence times among *Thalassiosira* spp. based on rDNA ITS1 sequence alignment. Time estimates are derived from a relaxed molecular clock calibrated using Sorhannus [12]. Branch numbers represent time of divergence (Mya). Chronogram shows that *T. rotula* split from *T. gravida* approximately 3.28 Mya. Within *T. rotula,* Lineages 1 and 2 split from Lineage 3 approximately 0.68 Mya. Lineages 1 and 2 diverged from one another approximately 0.22 Mya. The tree topology matches that of Sorhannus [12], with the divergence of *T. pseudonana* from all other *Thalassiosira* spp at 30 Mya. Placement of *T. weissflogii* and *T. guillardii* differ from Sorhannus [12], which may be due to differences in ITS1 and 18S mutation rates.

Using a dated phylogenetic analysis, divergence times were estimated for *T. gravida,* the three *T. rotula* lineages, and several other *Thalassiosira* species (Figure 4). Divergence between *T. gravida* and *T.rotula* was approximately 3.28 Mya. Lineage 3 was calculated to be the oldest lineage, from which lineages 1 and 2 diverged 0.68 Mya. Lineage 2 diverged most recently from lineage 1 at 0.22 Mya.

The ITS1 is present in multiple copies within the genome and thus each isolate may contain intra-genomic variants. Intra-genomic variation in the ITS1 was examined in one isolate from each lineage: VIA (lineage 1, site 2), PS (lineage 2, site 4), and SIS (lineage 3, site 6). From isolate VIA, 17 of 20 amplicons had sequences identical to sequence ID 1 (Table 3), and three were novel sequences that differed by 1bp. From isolate PS, 13 of 20 amplicons were identical to sequence ID 2, and the remaining 7 were novel singleton sequences that differed by 1–7 bps. From isolate SIS, 16 of 20 isolates were identical to sequence ID 3, and 4 represented novel sequences that each differed by 1bp. Average within-lineage sequence divergence was 0.18 ± 0.10%, 1.90 ± 0.50%, and 0.25 ± 0.18% for lineages 1, 2, and 3 respectively. Neither probabilistic divergence measures nor difference of sums of squares revealed recombination among sequence types (p>0.05). In addition, RDP3 analysis revealed no evidence for recombination among sequence types (p>0.05); however, with such low levels of divergence in this dataset, recombination may be below the limits of detection for RDP3 [63]. When added to the network analysis, intra-individual variants did not alter the initial network structure (data not shown) and lineage groupings 1–3 remained significant (p<0.001).

### Physiological variation

Triplicate maximum acclimated growth rates of axenic and xenic cultures of VIA were not significantly different (p>0.05) indicating that bacterial presence in cultures did not alter growth rate. Subsequent experiments were conducted using both xenic (CCAP1085_21, CCMP3264, VIB) and axenic (VIA, CCMP1647, SIS) isolates. Isolates VIA and VIB represented lineage 1 and were collected from the Eastern North Pacific (sites 2 and 3). Four isolates represented lineage 3, and were collected from the Western North Pacific (SIS, site 6), and the Mediterranean Sea (CCAP1085_21, CCMP1647, CCMP3264, sites 11, 12, and 15). No live isolates of lineage 2 were available.

**Figure 5:**
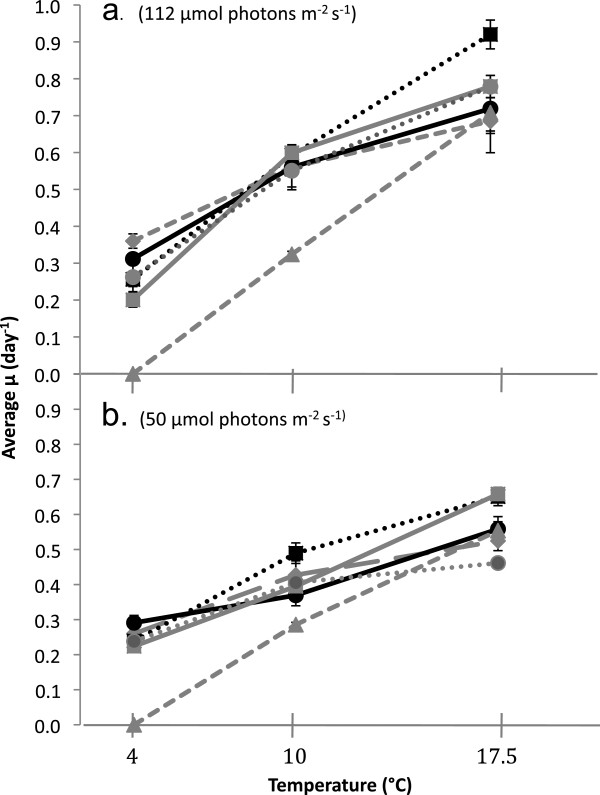
**Physiological Experiments.** Black lines represent lineage 1, grey lines represent lineage 3. **a**) Growth rates for six strains at 4, 10, and 17.5°C, high light (112 μmol photons m^-2^ s^-1^) **b**) Growth rates for six strains at 4, 10, and 17.5°C, low light (50 μmol photons m^-2^ s^-1^).

There was no significant clustering of growth rate with ITS1 lineage at any of the six light and temperature conditions (p>0.05). Instead, relative growth rate among isolates differed with treatment, illustrated by extensive crossing of growth curves as environmental conditions changed (Figures 5a and b). At high light intensity (112 μmol photons m^-2^ s^-1^), specific growth rates ranged from no growth to 0.92 ± 0.04 day^-1^ (Figure 5a). At low light intensity (50 μmol photons m^-2^ s^-1^), specific growth rates ranged from 0.22 ± 0.01 - 0.66 ± 0.01 day^-1^ (Figure 5b). At both light intensities, the CV was significantly larger at 4°C than at 10 or 17.5°C (p<0.05). Growth rate varied significantly among isolates at each temperature (p<0.05). There were no significant relationships between growth rate and cell diameter at any culturing condition (p>0.05).

The physiological response of isolates to light intensity was dependent on temperature. For example, at 4°C, there were no significant differences (p>0.05) in growth rates between high and low light intensities for all isolates except SIS (Figures 5a and 5b). In contrast, at 17.5°C growth rates differed significantly (p<0.05) between high and low light intensities for all isolates except VIB (Figures 5a and 5b).

### Variation in relative genome size

**Figure 6:**
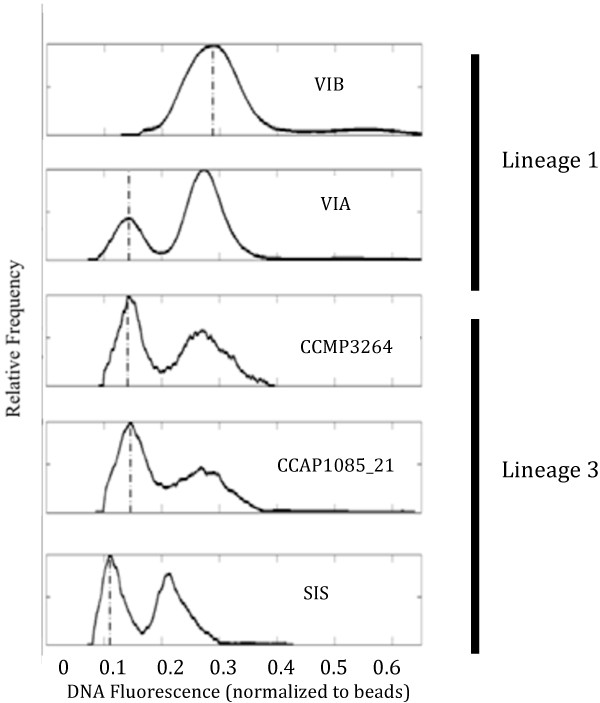
**Genome Size.** Average integrated fluorescence signal for five strains of *T. rotula* measured at G1 and G2 cell phases, measured using the IFCB. Fluorescence is normalized to 6um beads. Dotted line represents mode fluorescence of each strain in G1 cell phase.

Genome size was measured in five isolates. Two of these isolates represented lineage 1: VIA and VIB. Three isolates represented lineage 3: SIS, CCMP3264, and CCAP1085_21. No live isolates were available for lineage 2. The samples from all isolates contained cells in both G1 and G2 phases of the cell cycle, as indicated by bimodal distributions of integrated DNA fluorescence, with peaks separated by a factor of 2 in fluorescence intensity (Figure 6). G1 and G2 distributions provided an internal standard of our ability to detect changes in genome size. The ratio of G1:G2 cells in VIB was significantly higher than in other isolates (p<0.05), likely due to different physiological responses to light and temperature conditions used to culture cells for flow cytometry. Genome size, measured by DNA fluorescence, varied significantly among isolates. Among isolates from lineage 3, SIS exhibited 30% less G1 DNA fluorescence, at 0.104 relative fluorescence units (rfu) (p<0.05). The G1 DNA fluorescence of CCMP3264 and CCAP1085_21 was 0.15 rfu. Within lineage 1, the mode of G1 fluorescence signal of VIB was 0.27 rfu and was significantly different from all other isolates, with a nearly two-fold increase in fluorescence intensity at both G1 and G2 peaks (p<0.05).

There was no relationship between genome size and cell size measurements. Average cell volume of all isolates was 21.52 ± 5.56 μm^3^. There were no significant differences in cell volume between different isolates (p>0.05). However, VIB exhibited the largest average cell volume (28.65 ± 13.22 mm^3^), and exhibited the largest range in cell size.

## Discussion

To determine subdivision within the *T. rotula* morphospecies, it was first necessary to examine genetic divergence between *T. rotula* and *T. gravida*. Previous studies identified morphological plasticity in the characteristics used to define each species and argued for a single species designation [49,50]. Here, we found that culture collection isolates of the two species differed by at least 7% at the ITS1 and 0.8% at the 28S rDNA. This level of divergence is comparable to that observed between different species of the diatom *Skeletonema* (0.5% 28S divergence) [18] and between *Pseudo-nitzschia* species (7.2% ITS1 divergence) that were confirmed using mating experiments [15]. ITS1 sequence variation indicated that *T. rotula* and *T. gravida* diverged approximately 3.28 Mya. Furthermore, the predicted ITS1 secondary structures of the two species differed considerably, a characteristic related to reproductive incompatibility in protists [23-25,69]. Although the links between reproductive isolation and ITS1 folding structure are not as well understood as the ITS2, it has been suggested that the ITS1 and ITS2 molecules co-evolve to maintain important biochemical interactions necessary for processing the mature ribosome [26,70]. Here, differences in the predicted *T. gravida* and *T. rotula* folding structures indicate that significant evolution has occurred at the ITS1 [24,70]. Differences in rDNA sequences and predicted ITS1 secondary structures between culture collection isolates of *T. rotula* and *T. gravida* suggest that the original species designations are correct.

The majority of field isolates were not significantly different from *T. rotula* culture collection isolates at the 18S and 28S rDNA. This pool of isolates could be divided into three distinct ITS1 lineages which diverged from *T. rotula* culture collection isolates by 0–0.6%, an order of magnitude less than their divergence with *T. gravida*. This level of variation is comparable to that identified within other diatom species; for example, *Pseudonitzchia pungens* clades diverged by 0.5% at the ITS1 [71].

Several lines of evidence suggest that the three lineages may be able to interbreed. First, their predicted ITS1 secondary structures were identical to each other, suggesting that mutations in the ITS1 have not resulted in significant structural changes to this important molecule [70]. In addition, there were no compensatory base changes (CBCs) in the ITS1 stem regions of the three lineages, suggesting that this gene is conserved. Overall, the lack of CBCs and conservation of secondary structure among lineages suggest that they may retain the ability to interbreed. Second, there were no consistent differences in genome size among lineages, another indication that they may be able to interbreed. Importantly, differences in genome size were observed within and not between lineages. Within lineage 1, genome size differed by roughly two fold and within lineage 3, by 30%. Genome duplication, or polyploidization, is common in plants, and has been shown to result in rapid reproductive isolation [30,72-75]. In diatoms, few studies have examined changes in DNA content. Genome size differences of two-fold have been observed among ITS1 lineages of the diatom *D. brightwellii*, diverged by only 0.8% at the ITS1, suggesting that each lineage may instead represent a distinct species [28]. Furthermore, DNA content has been shown to vary among species within the genus *Thalassiosira,* suggesting that polyploidization may play a role in the evolution of diatoms as well as plants [29]. Because variations in genome size did not correlate with ITS1 lineage in *T. rotula*, this metric did not provide evidence for consistent barriers to interbreeding. Instead, the observation that two out of five strains differed in genome size suggests that *T. rotula,* and perhaps diatoms in general*,* may have a relatively plastic genome complement [29].

Although several lines of evidence suggest that interbreeding could occur, additional data suggest that these lineages may not be actively interbreeding. To look for signatures of recombination, multiple copies of the ITS1 were sequenced from individuals representing each lineage. If lineages were interbreeding, one might expect to find, for example, some copies or recombinants of a lineage 1 sequence in a lineage 2 individual [76]. No signature of recombination could be detected among lineages suggesting that interbreeding, if it occurs, is infrequent and below the threshold of detection. Here, we used a single genetic marker to examine recombination; future analysis of recombination would be improved by surveying a greater number of genes. It is also worth noting that diatoms divide primarily asexually, and that sexual cycles have been examined for only a handful of the large number of described species [77,78]. Sexual recombination in the field has been observed [79,80], but rarely, and estimates of the incidence sexual recombination in diatoms varies widely, from once per year to once every 40 yrs [81]. In addition to an inability to detect recombination, it appears that gene flow between lineages has been reduced for significant time periods. A dated phylogenetic analysis indicated that lineage 3 diverged from *T. gravida* 3.28 Mya. Lineages 1 and 2 diverged later, at 0.68 Mya. Because divergence calculations can vary depending on outgroups, genes, or calibration points used in analysis [12,82], divergence times should be interpreted cautiously. Even if these estimations are off by orders of magnitude, the estimated time since last interbreeding is significant.

In the marine environment, interbreeding could cease to occur through such mechanisms as isolation by distance, physical barriers to gene flow, competitive exclusion, environmental adaptation, or genetic and phenological characteristics that prevent gametes from fusing in the field [83]. Here, it appears that isolation by distance was an unlikely mechanism promoting differentiation among *T. rotula* lineages*.* For example, genetic distance among lineages was not related to geographic distance. In fact*, T. rotula* lineage 3 had a cosmopolitan distribution ranging from the Mediterranean Sea and the N. Atlantic to the N. Pacific. This distribution is comparable to that observed in lineages of the pennate diatom *P. pungens*[71] and contrasts with many terrestrial plant species, where genetic distance among lineages often correlates with geographic distance (eg. [84,85]). The observation that lineages can be broadly distributed in both centric and pennate diatoms suggests that dispersal likely plays a significant role in regulating gene flow. Lack of isolation by distance observed here suggests that there are no physical barriers impeding broad dispersal.

On smaller scales, physical features, such as water recirculation, may act to reduce gene flow, allowing different lineages to arise and be maintained. For example, hydrographic features have been hypothesized to drive genetic divergence in diatoms in coastal fjords of the NE Pacific where recirculating water may retain cells inside the fjord, allowing them to remain in and adapt to a particular location [55]. Interestingly, lineage 2 was observed within a recirculating coastal fjord in the NE Pacific, and exhibited significant divergence from lineage 1 sampled outside of the fjord, suggesting that water recirculation may influence genetic subdivision in multiple species of phytoplankton.

Competitive exclusion and environmental adaptation may be additional mechanisms initiating and supporting lineage divergence. Here, all but one location was dominated by just a single lineage. In those locations, the probability that other lineages were present but not detected was low. For example, a lineage representing 10% of the population would have a 99% probability of being detected in our 40 isolates collected from the N. Atlantic (Narragansett Bay and Martha’s Vineyard) [86], suggesting that these sites were likely dominated by single lineage. This may be due to competitive exclusion of lineages not adapted to the environment in Narragansett Bay and coastal N. Atlantic. There may, however, be more complex dynamics in play. For example, all three lineages were sampled from the Queen Charlotte Islands in the NE Pacific. This location may act as a hub of intermixing between water masses and may provide an environment heterogeneous enough to support the ecological niches of all three lineages.

To explore potential signatures of environmental adaptation, we compared the physiological response of each lineage to a range of light intensities and temperatures, two important environmental variables known to affect phytoplankton growth [87-89]. As established by Brand [90], differences in acclimated growth rates can be used to identify underlying genetic variation among isolates grown in a single environmental condition. Analyzing the growth rates of isolates under different conditions allows for comparisons of genotypic versus environmental effects [91]. If environment alone were driving differences in growth, isolates would exhibit the same relative difference in growth rate, regardless of environment. Here, there was no correlation between ITS1 lineage and growth characteristics examined. Instead, isolates exhibited significant genotype by environment [91] responses to light and temperature. The genotype by environment experiments conducted here suggest that there is additional clonal diversity within each *T. rotula* lineage, similar to that identified in other phytoplankton species [86,92,93]. Lack of clear differentiation between lineages in their physiological response to light and temperature does not mean that environmental adaptation has not occurred among lineages since other factors such as predation and nutrient availability may be more important drivers of environmental adaptation among lineages.

The persistence of single lineages within individual locations suggests that there may be yet another type of environmental adaptation that allows diatoms to diverge into distinct lineages. For example, all isolates collected from Narragansett Bay represented a single lineage, regardless of the month of sampling (January, February, June or October). Furthermore, isolates collected from the Gulf of Naples between 1993 and 2008 represented the same lineage, suggesting that it persisted over many years in a single habitat. Similarly, identical genotypes of *D. brightwellii* were detected in Puget Sound over a seven-year period [86]. The persistent occurrence of lineages at individual locations may relate to the ability of many diatoms to create resting spores, which lie dormant in the sediment and may remain viable for decades [94]. *T. rotula* can form resting spores, although it is not a required part of its life cycle and the frequency of resting spore formation in the field and the rate of germination success are unknown [95-98]. An intriguing hypothesis is that environmental adaptation to different phenological triggers for spore formation and germination could foster the initiation and maintenance of distinct lineages.

Finally, interbreeding could cease to occur among lineages if prezygotic barriers to gene flow no longer allow for sexual recombination among lineages. Prezygotic barriers to gene flow include changes to gametes that prevent them from recognizing each other [83]. In diatoms, the *Sig1* gene has been hypothesized to play an important role in gamete recognition [56]. This gene has undergone rapid evolution among strains of the diatom *T. weissflogii*, including distinct protein changes that may alter the interaction between gametes in the field [56]. This type of prezygotic barrier to gene flow may lead to speciation via reinforcement of postzygotic differentiation [76,79]. Previous attempts to amplify the *Sig1* gene in *T. rotula* have failed [56], but future transcriptional or genome-wide sequencing analyses may shed light on the potential for a prezygotic barrier to gene flow between lineages observed here. A prezygotic barrier to gene flow could well explain the conflicting data we obtained regarding interbreeding among lineages, where genome size and RNA secondary structure indicated no barriers to interbreeding but active recombination may no longer occur. In this scenario, prezygotic barriers to gene flow would prevent sexual recombination among lineages but the rDNA would not yet have diverged to levels that compare with more distantly-related species.

## Conclusions

Our data suggest that genetic divergence between *T. rotula* and *T. gravida* is significant and that they should continue to be described as distinct species, with future investigations to more fully describe differences between them. From a taxonomic standpoint, the divergence among *T. rotula* lineages is less clear. On one hand, it appears as if interbreeding between lineages has not occurred for long periods of time, suggesting that they could represent recently diverged cryptic species. On the other hand, lineages exhibited no differences in ITS1 secondary structure or genome size and exhibited no clear physiological partitioning, suggesting that they are conspecific. Prezygotic barriers to gene flow and differential adaptation to environmental factors involved in vegetative growth and/or spore formation are possible mechanisms that explain these conflicting results. Given the current data, the *T. rotula* lineages should be considered a single species. Future studies to tease apart their relationships will benefit from analyses of genetic variation beyond the rDNA.

The high dispersal marine environment is clearly not a barrier to speciation in diatoms, a group of organisms with an estimated 100–200,000 species, second only to angiosperms as the most diverse primary producers on the planet [11,13]. Confirming species identity in closely-related diatom species is particularly difficult because testing for reproductive compatibility in diatoms is often not feasible in the lab and ultimately, may not reflect recombination in the field [99]. Differences in the abundance of *T. rotula* lineages in the field suggest that past evolutionary events promoting their subdivision have led to lineage designations that are likely ecologically relevant, highlighting that in diatoms, a close interplay of ecology and evolution may regulate their impact on global biogeochemical cycles.

## Competing interests

This work is associated with no competing interests, financial or otherwise.

## Authors’ contributions

TR and KW conceived and designed the present study; KW, RO, and DR performed the experiments; KW and TR analyzed molecular data, RO and KW analyzed genome size data, DR, KW and TR analyzed physiological data; KW and TR wrote the manuscript. All authors read and approved the final manuscript.
